# Assisted partner notification services to augment HIV testing and linkage to care in Kenya: study protocol for a cluster randomized trial

**DOI:** 10.1186/s13012-015-0212-6

**Published:** 2015-02-13

**Authors:** Beatrice Muthoni Wamuti, Laura Kelly Erdman, Peter Cherutich, Matthew Golden, Matthew Dunbar, David Bukusi, Barbra Richardson, Anne Ng’ang’a, Ruanne Barnabas, Peter Maingi Mutiti, Paul Macharia, Mable Jerop, Felix Abuna Otieno, Danielle Poole, Carey Farquhar

**Affiliations:** Department of Research and Programs, Kenyatta National Hospital, Hospital Road, Upper Hill, Nairobi 00202 Kenya; Department of Pediatrics, University of Toronto, Hospital for Sick Children, 555 University Avenue, Toronto, ON M5G 1X8 Canada; NASCOP, Ministry of Health, Government of Kenya, Kenyatta National Hospital Grounds, P.O. Box 19361-00202, Nairobi, Kenya; Department of Epidemiology, University of Washington, 1959 NE Pacific Street, Health Sciences Building F-250, Seattle, WA 98195-7236 USA; Department of Medicine, University of Washington, 1959 NE Pacific Street, RR-512 Health Sciences Building, Seattle, WA 98195-6420 USA; Public Health–Seattle & King County HIV/STD Program, 401 5th Ave, Suite 1152, Seattle, WA 98104 USA; Center for Studies in Demography and Ecology, University of Washington, 206 Raitt Hall, Seattle, WA 98195-3412 USA; VCT and HIV Prevention Unit, Kenyatta National Hospital, Hospital Road, Upper Hill, Nairobi 00202 Kenya; Department of Global Health, University of Washington, 1510 NE San Juan Road, Seattle, WA 98195-7965 USA; Department of Biostatistics, University of Washington, F-600, Health Sciences Building, Seattle, WA 98195-7232 USA; Vaccine and Infectious Disease Division, Fred Hutchinson Cancer Research Center, 1100 Fairview Avenue North, Seattle, WA 98109 USA; Department of Global Health and Population, Harvard University, 655 Huntington Avenue, Boston, MA 02115 USA

**Keywords:** HIV, Assisted partner notification, Contact tracing, Linkage to care, HIV testing and counselling, Health advisors, Sub-Saharan Africa, Kenya

## Abstract

**Background:**

HIV case-finding and linkage to care are critical for control of HIV transmission. In Kenya, >50% of seropositive individuals are unaware of their status. Assisted partner notification is a public health strategy that provides HIV testing to individuals with sexual exposure to HIV and are at risk of infection and disease. This parallel, cluster-randomized controlled trial will evaluate the effectiveness, cost-effectiveness, and feasibility of implementing HIV assisted partner notification services at HIV testing sites (clusters) in Kenya.

**Methods/design:**

Eighteen sites were selected among health facilities in Kenya with well-established, high-volume HIV testing programs, to reflect diverse communities and health-care settings. Restricted randomization was used to balance site characteristics between study arms (*n* = 9 per arm). Sixty individuals testing HIV positive (‘index partners’) will be enrolled per site (inclusion criteria: ≥18 years, positive HIV test at a study site, willing to disclose sexual partners, and never enrolled for HIV care; exclusion criteria: pregnancy or high risk of intimate partner violence). Index partners provide names and contact information for all sexual partners in the past 3 years. At intervention sites, study staff immediately contact sexual partners to notify them of exposure, offer HIV testing, and link to care if HIV seropositive. At control sites, passive partner referral is performed according to national guidelines, and assisted partner notification is delayed by 6 weeks. Primary outcomes, assessed 6 weeks after index partner enrollment and analyzed at the cluster level, are the number of partners accepting HIV testing and number of HIV infections diagnosed and linked to care per index partner. Secondary outcomes are the incremental cost-effectiveness of partner notification and the costs of identifying >1 partner per index case. Participants are closely monitored for adverse outcomes, particularly intimate partner violence. The study is unblinded due to practical limitations.

**Discussion:**

This rigorously designed trial will inform policy decisions regarding implementation of HIV partner notification services in Kenya, with possible application to other parts of sub-Saharan Africa. Examination of effectiveness and cost-effectiveness in diverse settings will enable targeted application and define best practices.

**Trial registration:**

ClinicalTrials.gov NCT01616420.

## Background

HIV testing is central to HIV control. Knowledge of serostatus promotes safer sexual practices in a variety of populations [1-4] and is an essential first step in the uptake of HIV treatment, which improves survival and reduces HIV transmission [5]. However, despite increased awareness of HIV and improved availability of anti-retroviral therapy (ART), an estimated 60% of infected individuals in sub-Saharan Africa are unaware of their status [6].

Assisted partner notification services (APS) is a public health strategy used to curb the spread of sexually transmitted infections (STIs) by testing and treating the sexual partners of infected index cases. The intervention typically involves a public health worker interviewing persons diagnosed with an STI (i.e., index cases) about their sexual partner(s) and then providing the index case with some level of assistance notifying their partners and assuring their testing. Some health departments in the US and Europe developed APS programs for HIV as early as the 1980s, and APS has been found to increase HIV case-finding and safer behaviors [7]. Despite its resource-intensiveness, some studies suggest that APS can be cost-effective and even cost-saving due to the potential to prevent HIV transmission and the resulting costs of HIV care [8]. APS programs incorporate confidentiality safeguards and intimate partner violence (IPV) screening, and harms are rarely reported [7]. CDC guidelines now recommend APS integration into all HIV control programs [9].

US and European data supporting APS for HIV control must be cautiously applied to HIV-endemic regions. Differences in culture, socioeconomic conditions, health systems, background levels of HIV testing, the status of youth and women, and perceptions of HIV infection may all affect the feasibility and effectiveness of APS. Two recent reports from sub-Saharan Africa show promising results. An HIV APS program in Cameroon diagnosed one new case of HIV for every 3.2 index cases interviewed [10]. In a small (*n* = 245) randomized controlled trial at STI clinics in Malawi, APS doubled the number of sexual partners testing for HIV compared to usual practices, and >50% of these partners were HIV seropositive [11]. In both reports, APS was acceptable and safe. While these studies provide preliminary evidence that HIV APS can be productively implemented in sub-Saharan Africa, replication in large, well-designed trials is required before this strategy is widely adopted.

Kenya has an HIV prevalence of 5.6%. Despite major national initiatives to improve HIV testing, 53% of HIV-seropositive individuals are unaware of their status [12], and most perceive themselves to be at low or no risk [13]. Currently, there is no HIV APS in Kenya; the standard of care is passive referral, in which individuals testing HIV-seropositive are encouraged to disclose their status to their partners and bring them for testing. HIV APS has the potential to improve HIV control in Kenya by identifying those at risk and facilitating both testing and linkage to care. Kenya’s strong HIV research infrastructure provides the opportunity to answer relevant questions about scale-up by rigorously evaluating HIV APS. These factors brought the Kenyan Ministry of Health’s National AIDS and STI Control Programme (NASCOP), Kenyatta National Hospital (KNH), and the University of Washington together to conduct a cluster-randomized trial at HIV testing sites in Kenya to evaluate the effectiveness, cost-effectiveness, and feasibility of HIV APS across a variety of settings.

The primary objective of the study is to evaluate the effectiveness of HIV APS in Kenya for HIV case-finding and linkage to care among sexual partners of index cases. We hypothesize that APS will increase HIV testing, new HIV diagnoses, and linkage to care among HIV-positive partners compared to passive referral, and that case-finding will be enhanced by extending APS beyond the primary partner to other sexual partners. We also predict that HIV APS will be acceptable with minimal social harms, and that APS will be cost-effective from both payer and societal perspectives. A cluster-randomized design was selected to minimize risk of contamination between arms and to evaluate barriers in implementation and program costs at the facility level. Outcomes will be analyzed at the cluster level. Examining challenges and outcomes across diverse study sites will provide insights into best practices to help guide APS implementation.

## Methods

### Study design

This study is a multicenter, parallel, cluster-randomized controlled trial. In brief, 18 HIV testing sites in Kenya (clusters) were selected based on both practical and theoretical considerations and allocated to intervention and control arms (*n* = 9 clusters per arm) using a restricted randomization process (details below). In the intervention arm, APS is implemented immediately after index enrollment; in the control arm, APS is delayed for 6 weeks after index enrollment. Care provided in the control arm during the first 6 weeks of randomization is consistent with the current standard of care in Kenya (i.e., passive referral with no specialized partner services). Delayed provision of APS to the control arm allows for evaluation of additional study outcomes (i.e., partners notified and tested via APS >6 weeks after study enrollment), as well as providing these partners the opportunity for HIV testing. Sites enroll participants in a staggered fashion to ensure adequate time for individual site preparation and staff training, as well as oversight of study activities.

### Study sites

Eighteen HIV testing and counselling (HTC) sites in Kenya were selected as study sites (clusters). HTC in Kenya is carried out in a variety of settings by counsellors trained according to NASCOP guidelines [14]. Approaches include voluntary counselling and testing (VCT), provider-initiated testing and counselling (PITC), home-based testing, mobile testing, and antenatal testing. Treatment is provided by multidisciplinary teams at comprehensive care centers (CCC). This includes medical evaluation and management; laboratory monitoring; provision of antiretroviral treatment (ART) and co-trimoxazole; nutritional, adherence, and prevention counselling; and psychosocial support services [15].

**Figure 1:**
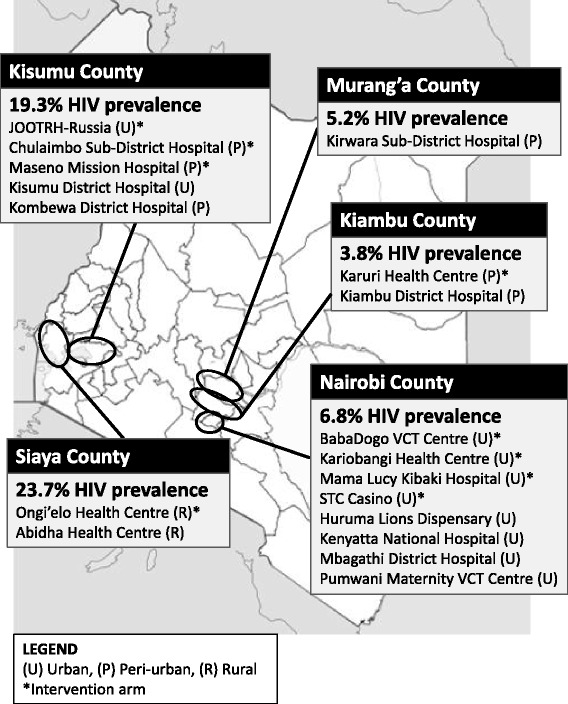
**Distribution of APS study sites in Kenya.** The 18 HIV testing and counselling (HTC) sites participating in the APS study are shown on a map of Kenya, with estimated HIV prevalence among adults for each county [16]. A star (*) indicates that the site was assigned to the intervention (immediate APS) arm. U, urban; P, peri-urban; R, rural.

Study sites were selected to reflect the diverse communities and health-care settings in Kenya in order to increase generalizability of results and to gain insights into implementing APS in different environments. As the first step in site selection, NASCOP identified >30 sites with well-defined VCT and/or PITC programs and moderate to high volume of HIV testing to facilitate timely completion of the study. Sites were then selected from this list to ensure diversity in terms of the following: geographical location within Kenya and by extension sociocultural practices; proximity to a city (urban, peri-urban, and rural); HIV prevalence; and health-care facility level. Principal investigators and study coordinators conducted site visits to obtain further details (e.g., the populations served, existing HTC and CCC services, space, rate of HIV case-finding). Based on these factors and consent for participation from facility leadership, they enrolled the final 18 sites (Figure 1).

### Randomization

**Figure 2:**
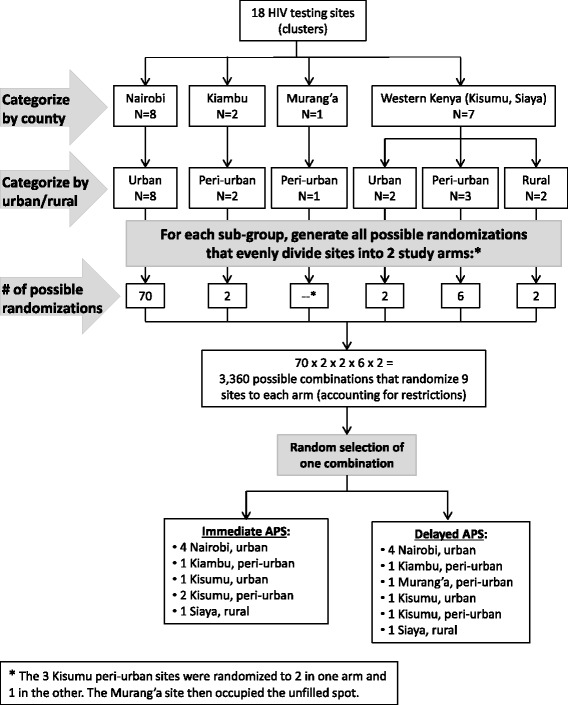
**Restricted cluster randomization of sites in APS study.** To ensure balance between study arms for key site characteristics, the 18 sites (clusters) were categorized by county and proximity to an urban area. Randomization was performed for each sub-group, and all possible combinations of these randomizations were generated. One combination was then selected using a random number generator. Note: the star (*) indicates that the three Kisumu peri-urban sites were randomized to two in one arm and one in the other; the Murang’a site then occupied the unfilled spot.

A restricted randomization process was used for site (cluster) allocation to intervention and control arms (Figure 2). To ensure balance between study arms in terms of key site characteristics, sites were categorized based on county (Nairobi, Kiambu, Murang’a, Kisumu) and proximity to a city (urban, peri-urban, rural). Within each sub-group, all possible randomizations that evenly distributed the sites into two study arms were generated using Excel; e.g., there were 70 ways to randomize the eight urban Nairobi county sites to four sites per arm. Since there were three peri-urban sites in Kisumu, they were randomized to two sites to one arm and one site to the other arm, and the Murang’a site was grouped with the single Kisumu site. These sub-group randomizations were then combined in all possible ways to distribute nine sites into each arm (*n* = 3,360), and one combination was selected using a random number generator.

Randomization and allocation were performed at the University of Washington (USA) by the study statistician, who had no knowledge of the sites other than the characteristics used for categorization. Cluster allocation was not concealed from the sites or study staff. To minimize bias, randomization was performed after enrollment of all clusters and prior to any individual enrollment.

Table 1 shows the baseline characteristics of the study sites. Distribution of geographic location and proximity to urban areas was comparable between study arms as a result of restricted randomization. Quantitative variables of interest—total and positive HIV tests per month, gender of clients, balance between VCT and PITC testing, and percent of clients testing positive—were similarly distributed in each study arm, with no significant differences by Mann–Whitney U test. Unfortunately, data on baseline rates of linkage to care are not routinely collected. Most of the selected facilities follow up with clients regarding linkage to care in some manner (by phone or home visits), and all have a CCC on site. The KNH VCT site also employs peer mentors (HIV-positive individuals enrolled in care) to improve linkage to care for newly diagnosed clients; no other sites routinely employed peer mentors or other strategies.

**Table 1:** **Baseline characteristics of APS study sites based on 2012 data.** ^**a**^

**Study arm**	**Study sites**	**County**	**Urban vs ** **rural**	**Facility funding source**	**Health facility level** ^**b**^	**Number of HTC staff**	**Number of HIV tests per month**	**Females among those tested (%)**	**Type of HTC used (%)**	**Number of positive HIV tests per month**	**Positive among tested (%)**	**Females among positive tests (%)**	**Start date at site**
Intervention: immediate APS	Kariobangi Health Centre	Nairobi	Urban	Nairobi City Council	2	2	170	61.1	VCT: 89.8	24	14.2	67.6	October 2013
									PITC: 10.2				
	BabaDogo VCT Centre	Nairobi	Urban	Nairobi City Council	2	2	212	46.4	VCT: 78.8	27	12.9	58.2	April 2014
									PITC: 21.2				
	STC Casino	Nairobi	Urban	Nairobi City Council	2	1	439	53.1	VCT: 51.1	43	9.9	55.9	July 2014
									PITC: 48.9				
	Mama Lucy Kibaki Hospital	Nairobi	Urban	Public	3	2	263	64.7	VCT: 64.1	38	14.3	64.0	July 2014
									PITC: 35.9				
	Karuri Health Centre	Kiambu	Peri-urban	Public	2	1	287	60.9	VCT: 75.3	13	4.5	61.3	June 2014
									PITC: 24.7				
	JOOTRH-Russia	Kisumu	Urban	Public	3	2	2,262	69.4	VCT: 2.4	134	5.9	68.8	August 2013
									PITC: 97.6				
	Maseno Mission Hospital	Kisumu	Peri-urban	Private	3	2	79	54.8	VCT: 40.5	6	7.7	58.9	March 2014
									PITC: 59.5				
	Chulaimbo Sub-District Hospital	Kisumu	Peri-urban	Public	3	3	551	55.2	VCT: 44.4	71	12.9	58.5	June 2014
									PITC: 55.6				
	Ongi’elo Health Centre	Siaya	Rural	Public	2	3	199	38.7	VCT: 28.2	15	7.6	59.7	June 2014
									PITC: 71.8				
Control: delayed arm	Kenyatta National Hospital	Nairobi	Urban	Public	4	7	3,942	60.3	VCT: 26.3	218	5.5	62.3	August 2013
									PITC: 73.7				
	Mbagathi District Hospital	Nairobi	Urban	Public	3	9	919	56.5	VCT: 13.4	106	11.6	55.4	November 2013
									PITC: 86.6				
	Huruma Lions Dispensary (NCC)	Nairobi	Urban	Nairobi City Council	2	1	435	76.7	VCT: 72.1	33	7.6	82.1	July 2014
									PITC: 27.9				
	Pumwani Maternity VCT Centre	Nairobi	Urban	Nairobi City Council	3	4	389	64.0	VCT: 100	16	4.1	65.8	July 2014
									PITC: 0				
	Kiambu District Hospital	Kiambu	Peri-urban	Public	3	2	778	55.0	VCT: 34.1	82	10.5	60.1	November 2013
									PITC: 65.9				
	Kirwara Sub-District Hospital	Murang’a	Peri-urban	Public	3	1	59	57.0	VCT: 18.3	3	5.0	65.7	June 2014
									PITC: 81.7				
	Kisumu District Hospital	Kisumu	Urban	Public	3	8	990	60.5	VCT: 23.1	129	13.1	58.9	November 2013
									PITC: 76.9				
	Kombewa District Hospital	Kisumu	Peri-urban	Public	3	4	293	52.9	VCT: 55.0	37	12.5	53.5	April 2014
									PITC: 45.0				
	Abidha Health Centre	Siaya	Rural	Public	2	2	141	67.4	VCT: 4.5	13	9.1	60.1	May 2014
									PITC: 95.5				

*Abbreviations*: *APS* assisted partner notification services, *HTC* HIV testing and counselling, *NASCOP* National AIDS and STI Control Programme, *PITC* provider-initiated testing and counselling, *VCT* voluntary testing and counselling.

^a^Data from the 2012 calendar year provided by the NASCOP Strategic Information Management Unit.

^b^Health-care facilities in Kenya are categorized into four levels: 1, community level; 2, sub-district facility; 3, district hospital; and 4, national hospital.

### Study procedures

#### Staff recruitment and training

We recruited NASCOP-certified HTC counselors with research experience to serve as APS study staff, henceforth referred to as ‘health advisors.’ Staff were trained and supported to provide high-quality APS by adapting training materials developed by the Cameroon Baptist Christian Health Board [10] to the Kenyan setting. Topics included are as follows: study procedures, client flow, informed consent processes, participant eligibility, notification of partners, IPV monitoring, data entry on smartphones, clinical training on HIV/AIDS staging, treatment and management, and diagnosis of opportunistic infections and sexually transmitted infections according to the Kenyan national guidelines. Refresher training sessions are conducted regularly, including updates on the new HTC algorithm introduced in 2013 by NASCOP [14].

#### Participants: inclusion/exclusion criteria

Inclusion criteria for index participants are defined as follows: 1) 18 years or older; 2) able to give informed consent; 3) HIV-seropositive test result at one of the study sites; 4) not enrolled in HIV care; and 5) willing to provide information on at least one sex partner from the preceding 3 years. Exclusion criteria are pregnancy (by self-report) and/or high risk for IPV due to safety concerns (described below). Partners are considered eligible for the study if 18 years or older and able to give informed consent.

#### Index participant enrollment

At study sites, clients are tested and counselled by resident HTC counsellors according to national HTC guidelines. At both intervention and control sites, all clients testing HIV-seropositive are informed about the APS study by the HTC counsellors (who are not employed by the study). If interested, clients are referred to health advisors for screening. Following a screening informed consent process, clients are screened for eligibility as an index participant. If eligible and willing to participate, written consent for study participation is obtained, and participants are informed whether they are in the immediate or delayed randomization arm.

Health advisors then administer questionnaires on demographic characteristics, sexual behavior, HIV testing history, and HIV care and treatment status. These questionnaires seek to elicit the following information on each of an index participant’s sexual partners from the prior 3 years: home/work addresses and contact information, index patient’s relationship to the partner (i.e., spouse, girlfriend or boyfriend, casual contact), and knowledge of the partner’s HIV status. Index participants are assigned unique identification numbers, and each partner is assigned a linked identification number.

#### Partner notification services

**Figure 3:**
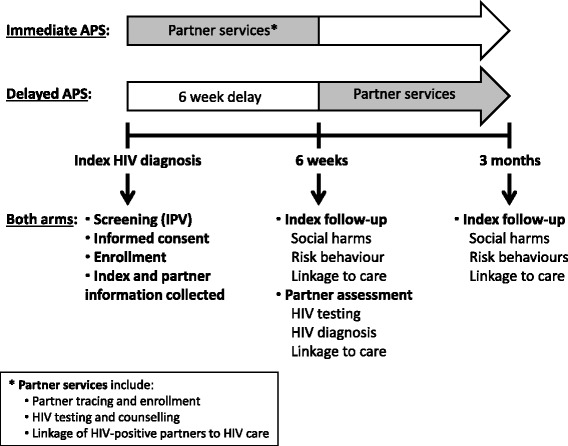
**Study flow for intervention versus control sites, starting from when an index participant tests HIV-seropositive.** Illustrated here are the study procedures for the intervention arm (immediate APS following index participant enrollment) and the control arm (APS delayed for 6 weeks after index participant enrollment). The star (*) indicates that the partner services involve: 1) partner tracing and enrollment; 2) HIV testing and counselling of partners; and 3) referral of HIV-seropositive partners to HIV care.

The timeline and components of partner notification in the intervention arm (immediate APS) and control arm (delayed APS) are displayed in Figure 3. At sites randomized to immediate APS, health advisors encourage index participants to disclose their HIV status to sexual partners and tell them that they will be contacting their partners within the next 1–2 weeks, consistent with provider referral [17]. At sites randomized to delayed APS, health advisors encourage index participants to disclose their HIV status to sexual partners (passive referral) as per HTC guidelines; health advisors then contact partners 6 weeks after index case enrollment.

Partners are first contacted via phone. To enhance credibility and create a safe space for the ensuing conversation, health advisors introduce themselves as Ministry of Health health-care workers based at KNH, the principal National Referral Hospital in Kenya. If partners ask how their contact information was obtained, they are told that it was obtained from a NASCOP database. In the original protocol, health advisors disclosed that a sexual partner had provided the contact information, without revealing the identity of the index participant. However, partners often declined to participate unless they knew who identified them. The script was changed after the first month to facilitate enrollment while protecting the confidentiality of index participants. This was the only change made to methods after trial commencement.

Health advisors then inquire whether the partner is in a suitable environment for health information to be discussed. If so, health advisors engage in a discussion about the partner’s health status, especially recent sexual contacts, risky sexual behavior and possible exposure to HIV. For individuals who are not in a suitable environment to discuss their health status, another phone call is planned or an in-person interview is scheduled.

On the phone or in person, health advisors encourage HIV testing and describe study objectives and the consent process. Partners are invited to speak with the health advisor and test for HIV at a study site close to their location. For those who cannot come to a study site, health advisors arrange a meeting at a convenient place and time; these have included partners’ homes, workplaces, or other public areas. When health advisors meet partners, they introduce themselves again, producing staff identification badges if necessary, and obtain written informed consent for the study. HTC is then performed. Health advisors provide HIV testing to those who agree, and HIV prevention counselling to those who decline. Confidentiality is emphasized throughout the process, and the partner is assured that their information will not be shared.

If a partner cannot be reached by phone after four attempts, the health advisor travels to his/her home or work and discreetly finds a way to speak to the partner in private so that confidentiality is maintained. They then describe the study as detailed above. If a partner still cannot be found after physical tracing, the health advisor verifies the contact information with the index participant and attempts to trace again. Failed physical tracing (at least three attempts), partners with insufficient contact information, and successfully traced partners who decline study participation are documented. Due to resource constraints, partners residing >50 km from the study site are notified via phone call, and if they cannot attend a study site for enrolment, they are not enrolled but still strongly encouraged to test at the nearest HTC facility.

#### Participant follow-up

Index cases participate in three study visits: one at baseline for screening/enrolment and two follow-up visits at 6 weeks and 3 months to assess linkage to HIV care, IPV and other social harms, and risk behaviors.

Partners in the immediate APS arm participate in two visits: one at baseline for recruitment and a follow-up visit after 6 weeks to determine whether they have tested for HIV and linked to care if HIV-seropositive. Linkage to care is verified by any of the following: CCC registration number, CD4 count, WHO HIV staging, or commencement of co-trimoxazole and/or ART. Partners in the delayed APS arm have a single visit scheduled at 6 weeks after enrollment of the index, which serves as a combined recruitment and assessment for any prior notification, HIV testing, and linkage to care if HIV-seropositive.

All participants are reminded via phone call on the day prior to a scheduled visit. Any participants who miss appointments are traced by health advisors (by phone, at home, or at work) and rescheduled. Any participants who cannot be traced at the closure of each study site (after approximately 6-months duration) are considered lost to follow-up.

#### Blinding

This study was carried out in an unblinded fashion. Blinding of study staff and participants was not feasible given the distinct protocols and timelines of the two study arms, including procedures for assessment of outcomes.

#### Data collection: smartphone technology

Quantitative data from both index and partner participants are collected by health advisors via smartphones using the Open Data Kit (ODK) platform. Pre-generated questionnaires programmed using ODK are downloaded from the NASCOP database and administered to participants. To optimize data consistency and accuracy at entry, completed questionnaires are uploaded over an encrypted connection to the NASCOP server immediately after the interview. Data verification and analysis are conducted weekly to ensure rapid, reliable data quality control and assurance.

#### Intimate partner violence monitoring

Due to concerns that HIV APS could lead to IPV for index cases, safeguards and monitoring for IPV were incorporated into the study protocol. IPV refers to physical, sexual, or psychological harm by a current or former partner or spouse, which can occur among heterosexual and same-sex couples [18]. We consulted IPV experts and community organizations regarding how to identify, counsel, and refer participants with a current or past history of IPV.Health advisors were trained according to a specific IPV screening protocol.

During screening, all potential participants are evaluated according to an IPV rating system that classifies them as low, moderate, or high risk. Individuals at high risk of IPV (reported IPV within the last 1 month) are not eligible for the study and are referred to IPV centers for counselling. We defined individuals at low risk IPV as: 1) no history of IPV during their lifetime either from a current or past partner; and 2) no fear of IPV if they participate in the study. These participants undergo standard study procedures and follow-up IPV case reports at 6 weeks and 3 months to capture any interim violence. Moderate risk IPV individuals were defined to have the following: 1) a history of IPV during their lifetime either from a current or past partner; and/or 2) fear of IPV if they participate in the study. Moderate risk participants receive additional special monitoring through scheduled phone calls or home visits at 10, 20, and 30 days after enrolment, as well as referral to IPV counselling and treatment centers identified *a priori* for further evaluation and management. Reports of IPV during the course of the study will be assessed on a case-by-case basis by the safety committee for further guidance. Internal monitoring for IPV is conducted through weekly data audits and IPV reports. The study safety committee, with representation from the study funder, conducts bi-annual meetings to monitor study progress and any reported IPV.

### Outcome measures

Outcomes will be compared between intervention and control arms based on data collected 6 weeks after enrollment of index participants and will be analyzed at the cluster level.

Primary outcomes are as follows: 1) HIV testing of partners (number of partners of an index participant that were tested for HIV, offset by the number of partners with locator information provided by the index participant); 2) newly identified HIV-infected partners (number of partners of an index participant identified as HIV-infected, offset by the number of partners of that index participant who were HIV tested); and 3) linkage to HIV care (number of partners of an index participant who were linked to HIV care, offset by the number of partners of an index participant identified as HIV-infected; analysis will be limited to index participants with at least one HIV-infected partner).

Secondary outcomes are as follows: 1) the incremental cost per HIV-infected person identified and linked to care, including ART, and the incremental cost-effectiveness per incident HIV infection, HIV-related death, and disability adjusted life-year (DALY) averted in Kenya from payer and societal perspectives; and 2) costs of identifying >1 partner per index case.

There have been no changes to outcome measures.

### Sample size and power analysis

At least 1,080 index participants will be enrolled across 18 HIV testing sites (clusters) with a minimum of 60 index cases per site. Sample size was calculated to ensure adequate power to address the study’s primary hypothesis: that immediate APS will increase the number of partners testing for HIV compared to delayed APS. Calculations are based on the assumptions that cluster sizes will be similar and that study sites will be distributed similarly between arms due to the restricted randomization procedure, which will increase face validity of the trial and decrease site-to-site variation across arms. The number of partners tested for HIV per index case is assumed to be Poisson (mean = *λ*_0_), and the coefficient of variation from site-to-site is assumed to be *k*. We have conservatively estimated *k* = .25. We also assume that an average between one and two partners per site will test for HIV in the delayed arm sites before 6 weeks (−0.2). Finally, we have assumed that the intervention will increase the outcome by a minimum factor *r* = 2 (twofold difference). Using an adapted Hayes and Moulton formula and conservative estimates (*k* = 0.25, *λ*_0_ = 0.1, *α* = 0.05 (two-tailed), with power = 0.80), if 60 participants are enrolled at each site in each arm, this requires nine sites per arms to see a twofold difference between the intervention and delayed arms.

### Statistical methods and analysis

For the primary outcomes, generalized estimating equations models with a Poisson link will be used to compare intervention and control arms at 6 weeks post-enrollment of index participants. The use of these models will allow analysis of data at the cluster level.

Cost-effectiveness analyses will follow the WHO guidelines [19] and use mathematical models to simulate the health outcomes from study data and to consider clinical outcomes beyond the scope of the study. Using the outcomes and costs from the study and effectiveness (health outcomes) estimated by the model, the cost-effectiveness will be estimated. To estimate effectiveness of APS compared to existing passive partner notification, a compartmental, deterministic disease progression and transmission model for HIV based on previous HIV and STI models will be used [20]. The model will be parameterized by data from population-based studies in Kenya and the literature and validated using Kenyan demographic health survey and antenatal data. The model will be used to predict the potential impact of APS on HIV incidence, progression, and mortality. Sensitivity and scenario analyses will account for uncertainty in parameters including varying impact of APS on identifying new HIV cases. Incremental costs for the intervention and treatment costs incurred (and averted) as a result of the intervention will be estimated in comparison to existing passive partner notification and HIV-care costs. Using these estimates of APS effectiveness and incremental cost of APS per HIV-positive person identified and treated, the incremental cost-effectiveness ratio per incident HIV case, HIV-related death, and DALY averted will be calculated.

### Ethics statement

This trial was approved by the Kenyatta National Hospital/University of Nairobi Ethics Review Committee (P523/10/2012) and the University of Washington Human Subjects Institutional Review Board Committee (43628 C). All participants in the study provided written informed consent. The trial is registered at ClinicalTrials.gov (NCT01616420).

### Trial status

Implementation of the study began in August 2012. At the time of submission, the intervention had been introduced to all 18 sites; data collection is ongoing.

## Discussion

Provision of partner services in the US and Europe has been shown to be safe and effective, as well as cost-effective for HIV case-finding and linkage to care [7,8]. Prior to launching similar programs in sub-Saharan Africa, it is important to evaluate HIV APS in rural and urban African settings, which have different HIV prevalences, affected populations, sociocultural milieus, and health system structures and resources. This study is the first cluster-randomized trial to evaluate HIV APS in sub-Saharan Africa and builds upon prior work in Cameroon and Malawi.

In Cameroon, evaluation of an HIV APS program (*n* = 1,462 indexes) showed that it was feasible to notify partners (60% of named partners), identify new HIV cases (50% of partners who tested), and link them to care (85% of those testing positive) [10]. However, APS in Cameroon was undertaken as a public health program within a large, faith-based organization providing medical care rather than a research study. Evaluation of the program was uncontrolled, did not document the proportion of persons who accepted the intervention, and did not include systematic long-term participant follow-up to evaluate clinical outcomes or adverse events. Thus, while the Cameroonian experience demonstrates that APS is scalable in at least one setting in sub-Saharan Africa and suggests that the intervention is effective, it does not provide the sort of evidence produced by a randomized trial. A well-designed individual-level randomized controlled trial in Malawian STI clinics [11] showed that APS was acceptable to index cases (89% of those eligible enrolled) and doubled HIV testing among partners (51% in APS versus 24% in patient referral arm). Sixty-four percent of those tested were HIV-seropositive, demonstrating that APS enriches for HIV case-finding, but the applicability of these results to lower-risk and rural populations is unclear. Our study addresses these gaps, and a randomized design ensures rigorous comparison between intervention and control arms.

This study is designed to demonstrate whether APS is more effective than passive referral for identifying individuals not previously aware of their HIV positive status. We expect this to be the case based on consistent increases in HIV case-finding associated with APS across populations and continents [7,10,11]. In African studies, the rate of HIV-positive cases among partners was greater than tenfold the national prevalence [6,10,11]. Linkage to care in the context of APS has been minimally investigated [10,21], and this study may contribute important data on whether APS can link partners to care and treatment.

HIV APS in Kenya also has the potential to be cost effective. A decision-analysis model based on the Malawian APS data [22] found that compared to passive referral APS reduced new transmissions by 12% with an incremental cost-effectiveness ratio (from a health system perspective) of $4,106 (USD) per transmission averted. The analysis may have underestimated cost-effectiveness, since it considered only 1 year of potential transmission and did not account for payer costs related to opportunistic infections (e.g., hospitalizations). On the other hand, the analysis may also have overestimated the intervention’s cost-effectiveness because it assumed that a single health advisor could work a large number of cases per year, which probably exceeds what is possible in actual practice. The cost of APS for each new HIV diagnosis was higher than that for passive referral ($36 versus $8) but lower than the cost of HTC strategies in Uganda ($43–231 per new diagnosis) [23]. There are differences between Malawi and Kenya, including a higher HIV prevalence in Malawi (10.8% versus 5.6% [24]). Interestingly, at lower HIV prevalence in the context of poor awareness of HIV status (as in Kenya), APS may be more cost-effective than VCT since individuals are less likely to suspect infection and seek testing [25]. HIV prevalence varies widely within Kenya, and cost-effectiveness will likely vary under different conditions. Combined with data on APS effectiveness from specific sites, we can identify facilities where introducing and/or scaling up APS would be most beneficial from the public health and Ministry of Health perspectives.

In terms of study execution, we have found that health advisor personalities and training are critical for the success of APS. Previous reports have found that individuals decline HIV APS due to shock and shame about HIV diagnosis, mistrust of public health services, and fear of notifying partners [11,26]. APS study health advisors were selected based on experience as effective HCT counselors and received additional training on how to build rapport, assure confidentiality, and convey potential benefits for index participants and partners. Health advisors report that index participants often name only one partner but may reveal additional partners after tactful probing. As we hypothesize that tracing multiple partners will increase HIV case-finding, health advisors are encouraged to share non-judgmental approaches to enhance disclosure of multiple partners, while respecting autonomy. Such approaches would be important to incorporate into an APS program. We are also planning a qualitative study to explore why individuals in Kenya may decline to participate in APS and how such barriers may be overcome.

Challenges have arisen during the partner notification process. Initially, partners may be resistant to communication with health advisors due to suspicions about the identity of the caller (e.g., prank caller, debt collectors). These concerns are usually assuaged by producing proof of identification and affiliation with the Ministry of Health-NASCOP and KNH. As described, the script for partner notification was modified early in the study to avoid partners demanding to know who provided their information; this has improved acceptance. Another challenge for health advisors is verbal abuse from partners during phone calls. Though they have never encountered physical violence, staff are expected to follow safety precautions such as travelling in pairs to risky or remote areas. Finally, enrollment of notified partners has been difficult at some sites. Success likely depends on the approach of health advisors, partner populations, and the interaction between them. Exploring how effectiveness varies by site characteristics and health advisor may highlight best practices.

We have also encountered health system challenges during the study. Kenya experiences shortages of HIV diagnostic test kits, and this has impeded enrollment at some sites more than others. At some facilities, CD4 testing was temporarily unavailable due to flow cytometer breakdown, hindering fulfillment of linkage-to-care criteria. While these resource limitations are reflective of the reality on the ground, they may skew measurement of intervention effectiveness given the 6-week time constraint for partner tracing and linkage to care and depending on which sites in each study arm are affected. They may also inflate the calculated costs of APS by prolonging the time required for enrollment, tracing, and testing. We have attempted to minimize such effects by sharing test kits between sites, ordering kits directly from suppliers when necessary, and shipping samples to other laboratories for CD4 testing. Our analyses will require interpretation in light of these systemic issues.

Active monitoring for IPV among index cases is ongoing, given the close association between HIV and IPV [27,28]. Baseline screening has identified both women and men experiencing IPV, and these cases were handled as per protocol. Similar to published literature [7,10,11], we have not detected any new or worsening IPV due to APS. Occasionally, APS has caused other social harms, such as relationship dissolution. Developing strategies to support individuals will be necessary if APS is scaled-up.

### Study limitations

There may be limitations to this study in terms of representativeness. We selected study sites to reflect the geographic, socioeconomic, and cultural diversity in Kenya. However, practical factors such as space constraints or testing volumes excluded certain facilities.

At the index participant level, certain groups may be underrepresented, as those reluctant to participate may decline referral to health advisors. For example, individuals facing stigma related to their partners—e.g., men having sex with men, commercial sex workers—may be less likely to participate for fear of discrimination. We attempted to mitigate this via staff sensitivity training and by including a site that primarily serves sex workers; however, a different study design would be required to fully address this question in key affected populations. Individuals hospitalized with advanced HIV disease and diagnosed through PITC may be excluded due to inability to consent, even though their partners are highly likely to benefit from APS.

At the partner participant level, some partners who were meant to be followed up after 6 weeks were instead brought in by the index partner immediately for notification, making it difficult to maintain site integrity. Moreover, it is difficult to trace partners who are homeless, lack reliable phone access or contact information, or reside in inaccessible regions. We excluded partners >50 km away from the study site due to resource constraints, though a nationwide program could overcome this through a health advisor network. The other issues will require solutions that balance cost-effectiveness with equity for vulnerable populations.

Some aspects of our study may be difficult to translate to a program level. The use of smartphones for data input and transmission to a central database has increased efficiency and portability and likely avoided transcription errors. However, these devices are relatively expensive and in the absence of widespread electronic medical records in Kenya, it is unclear how feasible and cost-effective this would be for scale-up of HIV APS. They may be more cost-effective in the long run compared to computers that require a power source and security measures. Finally, while blinding was not feasible with this study design, and most outcomes are objective, lack of blinding could contribute to biases, such as how intensely health advisors pursue tracing or encourage HIV testing.

## Conclusions

In summary, this large cluster-randomized trial will provide critical data on the effectiveness, cost-effectiveness, and feasibility of HIV APS in sub-Saharan Africa and facilitate rational, targeted application of APS in Kenya and potentially diverse environments in Africa. Trial implementation would not be feasible without the longstanding collaboration between KNH, NASCOP, and University of Washington, all organizations with strong commitments to investigate the utility of APS as part of Kenya’s HIV control strategy. HIV APS experts in Cameroon and the US also provided invaluable guidance regarding study design, health advisor training, program implementation, and IPV monitoring. Kenyan health officials and academics will continue to collaborate in order to apply study results within the health system. Our investigation may uncover concerns unique to the Kenyan setting and will inform programmatic implementation of HIV APS if this trial demonstrates effectiveness.

## Abbreviations

ART: Antiretroviral therapy
APS: Assisted Partner Notification Services
CCC: Comprehensive Care Center
DALY: Disability adjusted life-year
HIV: Human immunodeficiency virus
HTC: HIV testing and counselling
IPV: Intimate partner violence
KNH: Kenyatta National Hospital
NASCOP: National AIDS and STI Control Programme
ODK: Open Data Kit
PITC: Provider-initiated testing and counselling
VCT: Voluntary counselling and testing

## References

[1] Farquhar C, Kiarie JN, Richardson BA, Kabura MN, John FN, Nduati RW (2004). Antenatal couple counseling increases uptake of interventions to prevent HIV-1 transmission. J Acquir Immune Defic Syndr..

[2] Fonner VA, Denison J, Kennedy CE, O’Reilly K, Sweat M (2012). Voluntary counseling and testing (VCT) for changing HIV-related risk behavior in developing countries. Cochrane Database Syst Rev..

[3] Marks G, Crepaz N, Janssen RS (2006). Estimating sexual transmission of HIV from persons aware and unaware that they are infected with the virus in the USA. AIDS..

[4] Marks G, Crepaz N, Senterfitt JW, Janssen RS (2005). Meta-analysis of high-risk sexual behavior in persons aware and unaware they are infected with HIV in the United States: implications for HIV prevention programs. J Acquir Immune Defic Syndr..

[5] Cohen MS, Chen YQ, McCauley M, Gamble T, Hosseinipour MC, Kumarasamy N (2011). Prevention of HIV-1 infection with early antiretroviral therapy. N Engl J Med..

[6] UNAIDS: Global HIV/AIDS response: epidemic update and health sector progress towards universal access: progress report 2011. http://www.unaids.org/sites/default/files/media_asset/20111130_UA_Report_en_1.pdf. Accessed 1 March 2014.

[7] Hogben M, McNally T, McPheeters M, Hutchinson AB (2007). The effectiveness of HIV partner counseling and referral services in increasing identification of HIV-positive individuals a systematic review. Am J Prev Med..

[8] Varghese B, Peterman TA, Holtgrave DR (1999). Cost-effectiveness of counseling and testing and partner notification: a decision analysis. AIDS..

[9] National Center for HIV/AIDS, Viral Hepatitis, STD, and TB Prevention (US), Division of STD Prevention, Centers for Disease Control and Prevention (US) (2008). Recommendations for partner services programs for HIV infection, syphilis, gonorrhea, and chlamydial infection. MMWR Recommendations and Reports: Morbidity and Mortality Weekly Report. Recommendations and reports/centers for disease control.

[10] Henley C, Forgwei G, Welty T, Golden M, Adimora A, Shields R (2013). Scale-up and case-finding effectiveness of an HIV partner services program in Cameroon: an innovative HIV prevention intervention for developing countries. Sex Transm Dis..

[11] Brown LB, Miller WC, Kamanga G, Nyirenda N, Mmodzi P, Pettifor A (2011). HIV partner notification is effective and feasible in sub-Saharan Africa: opportunities for HIV treatment and prevention. J Acquir Immune Defic Syndr..

[12] NASCOP Kenya AIDS indicator survey. 2012. http://nascop.or.ke/3D/simupubs.php. Accessed 10 March 2014.

[13] Ng’ang’a A, Waruiru W, Ngare C, Ssempijja V, Gachuki T, Njoroge I (2014). The status of HIV testing and counseling in Kenya: results from a nationally representative population-based survey. J Acquir Immune Defic Syndr..

[14] NASCOP. HTC publications. 2014. http://nascop.or.ke/htcpubs.php. Accessed 23 March 2014.

[15] NASCOP. Antiretroviral therapy publications: care and treatment, basic package of support and care. 2014. http://nascop.or.ke/artpubs.php. Accessed 12 April 2014.

[16] NASCOP. Kenya HIV prevention revolution road map. 2014. http://www.nacc.or.ke/attachments/article/418/Kenya_HIV_Prevention_Revolution_Road_Map.pdf. Accessed 2 December 2014.

[17] Holmes KK, Sparling PF, Stamm WE, Piot P, Wasserheit JH, Corey L (2008). Sexually transmitted diseases.

[18] Centers for Disease Control and Prevention. Intimate partner violence. 2014. www.cdc.gov/violenceprevention/intimatepartnerviolence/index.html. Accessed 1 April 2014.

[19] UNAIDS. Costing guidelines for HIV prevention strategies. 2000. http://data.unaids.org/publications/irc-pub05/jc412-costguidel_en.pdf. Accessed 4 May 2014.

[20] Barnabas R, Ying R, van Rooyen H, Murnane P, Hughes J, Baeten J (2013). Use of HIV viral-load suppression to estimate the effect of community-wide home-based HIV counselling and testing and linkage to antiretroviral therapy on HIV incidence in South Africa: a mathematical modelling analysis. Lancet..

[21] Bocour A, Renaud TC, Udeagu CC, Shepard CW (2013). HIV partner services are associated with timely linkage to HIV medical care. AIDS..

[22] Rutstein SE, Brown LB, Biddle AK, Wheeler SB, Kamanga G, Mmodzi P (2014). Cost-effectiveness of provider-based HIV partner notification in urban Malawi. Health Policy Plan..

[23] Menzies N, Abang B, Wanyenze R, Nuwaha F, Mugisha B, Coutinho A (2009). The costs and effectiveness of four HIV counseling and testing strategies in Uganda. AIDS..

[24] UNAIDS. Country data for HIV. 2013. www.unaids.org/en/regionscountries/countries. Accessed 13 April 2014.

[25] Armbruster B, Helleringer S, Kalilani-Phiri L, Mkandawire J, Kohler HP (2011). Exploring the relative costs of contact tracing for increasing HIV case finding in sub-Saharan countries. J Acquir Immune Defic Syndr..

[26] Edelman EJ, Cole CA, Richardson W, Boshnack N, Jenkins H, Rosenthal MS (2014). Opportunities for improving partner notification for HIV: results from a community-based participatory research study. AIDS behav.

[27] Li Y, Marshall CM, Rees HC, Nunez A, Ezeanolue EE, Ehiri JE (2014). Intimate partner violence and HIV infection among women: a systematic review and meta-analysis. J Int AIDS Soc..

[28] Phillips DY, Walsh B, Bullion JW, Reid PV, Bacon K, Okoro N (2014). The intersection of intimate partner violence and HIV in U.S. women: a review. J Assoc Nurses AIDS Care.

